# Supplementary material for: A Novel Approach for Detecting Unique Variations among Infectious Bacterial Species in Endocarditic Cardiac Valve Vegetation
Source: Cells. 2020 Aug 13;9(8):1899. doi: 10.3390/cells9081899 (PMC7464176; doi:10.3390/cells9081899)
Supplement: Supplementary file 1 [file cells-09-01899-s001.pdf]

cells

MDPI

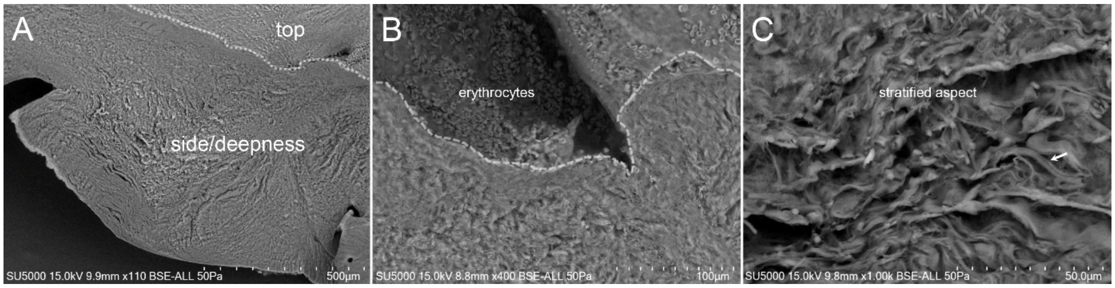

Figure S1. Scanning electron microscopy of control uninfected valve. **(A)** Low magnification view showing the surface and the depth of the valve along the dissection-cutting plane. (**B**) Erythrocytes located on the surface of the valve, which presented a stratified matrix along its depth. (**C**) High magnification view showing the layered organization with bundles of collagen-like or elastin-like appearance (arrow). No bacteria were observed.

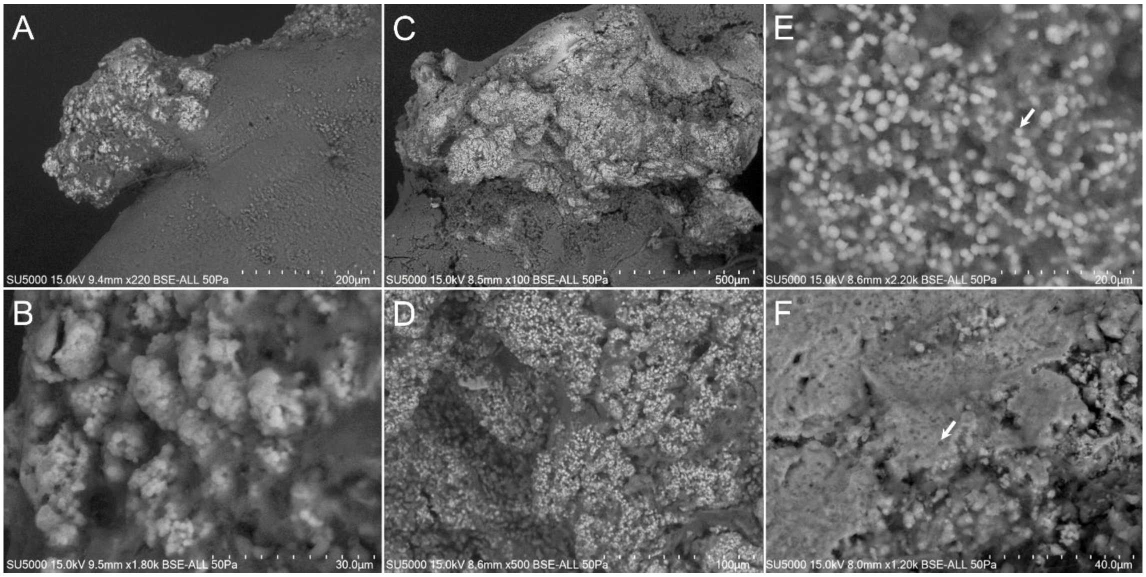

Figure S2. Scanning electron microscopy of *S. gallolyticus* infected vegetation showing bright regions. **(A–D)** Low magnification views of the bright regions, from the periphery of the vegetation to its center. (**E**) High magnification showing single bacteria or bacteria in chains. (**F**) Holes with bacteria ghost shapes in an amorphous matrix of a bright region.

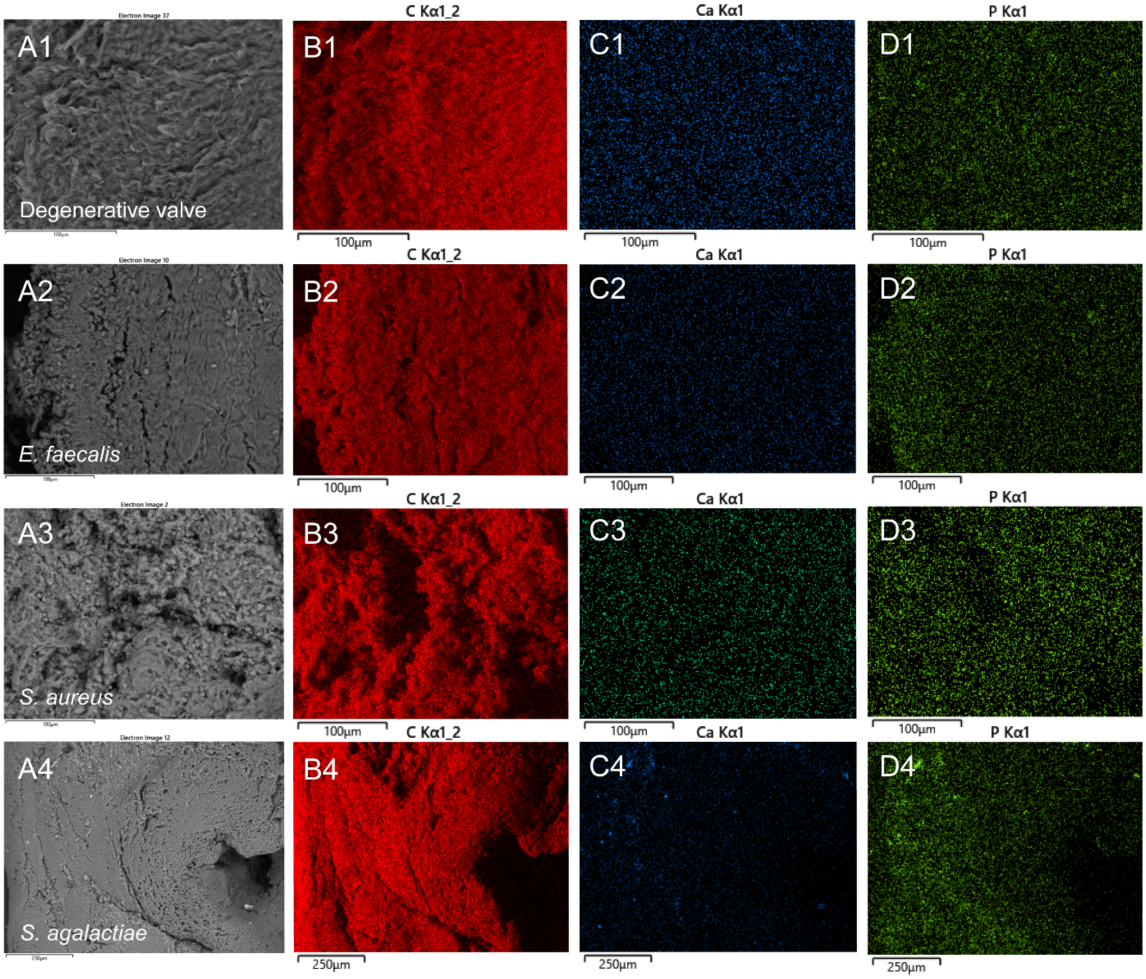

Figure S3. Scanning electron microscopy coupled to energy-dispersive X-ray (EDX) spectroscopy of representative regions from the degenerative control valve and *E. faecalis*, *S. aureus* and *S. agalactiae* infected vegetations. Degenerative valve, (2) *E. faecalis*, (3) *S. aureus*. (4), *S. agalactiae* vegetation. (**A**)SEM view. (**B**–**D**) Multi-element EDX mapping images of C, Ca and P.

Table S1. Weight and atomic percentage of C, O, N, P and Ca in the bright and non-bright regions of the *S. oralis* vegetation.

**Weight% non-bright zone**					**Weight% bright zone**					
**C**	**O**	**N**	**Ca**	**Ca/P**	**C**	**O**	**N**	**Ca**	**P**	**Ca/P**
57,05	26,36	14,74	0,27	0,22	51,58	30,81	13,81	2,61	1,29	2,02
57,57	27,38	14,81	0,12	0,12	50,52	28,63	17,3	1,59	0,83	1,92
51,81	25,55	20,84	0,6	0,33	49,16	28,17	15,24	3,95	1,77	2,23
55,63	24,82	16,72	0,71	0,33	53,88	26,69	14,45	2,98	1,41	2,11
Mean weight% non-white zone					Mean weight% white zone					
**55,52**	**26,03**	**16,78**	**0,43**	**0,25**	**51,29**	**28,58**	**15,2**	**2,78**	**1,33**	**2,07**
Atomic% non-bright zone					Atomic% bright zone					
**C**	**O**	**N**	**Ca**	**Ca/P**	**C**	**O**	**N**	**Ca**	**P**	**Ca/P**
63,58	22,05	14,08	0,09	0,09	58,96	25,96	13,54	0,89	0,57	1,56
63,33	22,61	13,97	0,04	0,05	57,57	24,5	16,9	0,54	0,37	1,46
58,05	21,49	20,03	0,2	0,14	57,56	24,76	15,3	1,39	0,8	1,74
62,44	20,91	16,09	0,24	0,14	61,34	22,81	14,11	1,02	0,62	1,65
Mean atomic% non-white zone					Mean atomic% white zone					
**61,85**	**21,77**	**16,04**	**0,14**	**0,11**	**58,86**	**24,51**	**14,96**	**0,96**	**0,59**	**1,6**
